# Preparation, Characterization and Mechanical Properties of Bio-Based Polyurethane Adhesives from Isocyanate-Functionalized Cellulose Acetate and Castor Oil for Bonding Wood

**DOI:** 10.3390/polym9040132

**Published:** 2017-04-05

**Authors:** Adrián Tenorio-Alfonso, María Carmen Sánchez, José M. Franco

**Affiliations:** 1 Department of Chemical Engineering, University of Huelva, Campus El Carmen, Campus ceiA3, 21071 Huelva, Spain; adrian.tenorio@diq.uhu.es (A.T.-A.); mcarmen@uhu.es (M.C.S.); 2 Pro^2^TecS-Chemical Process and Product Technology Research Center, University of Huelva, 21071 Huelva, Spain

**Keywords:** biosourced adhesives, castor oil, cellulose acetate, isocyanate, polyurethane, rheology

## Abstract

Nowadays, different types of natural carbohydrates such as sugars, starch, cellulose and their derivatives are widely used as renewable raw materials. Vegetable oils are also considered as promising raw materials to be used in the synthesis of high quality products in different applications, including in the adhesive field. According to this, several bio-based formulations with adhesion properties were synthesized first by inducing the functionalization of cellulose acetate with 1,6-hexamethylene diisocyanate and then mixing the resulting biopolymer with a variable amount of castor oil, from 20% to 70% (wt). These bio-based adhesives were mechanically characterized by means of small-amplitude oscillatory torsion measurements, at different temperatures, and standardized tests to evaluate tension loading (ASTM-D906) and peel strength (ASTM-D903). In addition, thermal properties and stability of the synthesized bio-polyurethane formulations were also analyzed through differential scanning calorimetry and thermal gravimetric analysis. As a result, the performance of these bio-polyurethane products as wood adhesives were compared and analyzed. Bio-polyurethane formulations exhibited a simple thermo-rheological behavior below a critical temperature of around 80–100 °C depending on the castor oil/cellulose acetate weight ratio. Formulation with medium castor oil/biopolymer weight ratio (50:50 % wt) showed the most suitable mechanical properties and adhesion performance for bonding wood.

## 1. Introduction

In the 21st century, sustainable development has become one of the main objectives in industrial activity. The approaching exhaustion of petroleum supplies, their fluctuating and rising prices, along with the stringent regulations as a consequence of the increasing environmental concerns have propelled scientists towards using renewable natural resources in order to replace total or partially petroleum-based raw materials, with the aim of overcoming these issues [1,2].

In the adhesive field, traditional formulations are petro-based [1,3], containing some volatile organic compounds (VOC) and other toxic substances, such as formaldehyde derivatives. Those materials are hazardous to the environment and detrimental to the human health, which encourages the adhesive industry to develop adhesives from renewable raw materials with suitable functional properties [4,5]. The use of eco-friendly alternatives in this field can help to overcome those problems. In fact, some natural adhesives like starch, casein and other proteins were used at the start of the 20th century [6]. Nevertheless, their utilization is limited as they exhibit low durability and low water resistance. In the past few years, as recently reviewed by Ferdosian et al. [7], some bio-based adhesives have been developed from a range of natural resources, including lignin [8], starch [1], tannin [9], vegetable oils [3], soy flour and soy protein [4]. However, there are still some properties to be sharpened to make those natural-based adhesives competitive in comparison to the traditional ones [10]. In this sense, bio-based polyurethanes seem to be an excellent alternative to petroleum-based products, overcoming the environmental drawbacks but, at the same time, providing suitable properties.

Since their discovery by Otto Bayer and co-workers in 1937 [11], polyurethanes have become one of the most outstanding polymers as they exhibit a high performance and versatility, being employed in a vast range of industrial and engineering applications such as foams [12,13], coatings [14], medicinal products [15,16] or adhesives [17], among others. This wide variety of applications is associated to their superior properties, including excellent corrosion, solvent and chemical resistance, high mechanical strength, low temperature flexibility, adhesion, suitable curing rates, chemical structure versatility, etc. [18,19,20]. As is well known, polyurethanes are polymers containing urethane linkages (NHCOO) in the main polymer chain [21]. In most cases, the synthesis of polyurethanes is accomplished through the reaction taking place between isocyanates (NCO) and active hydroxyl groups (OH) and using a chain extender (low molecular weight glycol or amine) [2,3,19]. Typical polyurethanes are multiblock copolymers whose structure comprises two domains, called “soft” and “hard” segments, which provide them with their characteristic exceptional versatility [22,23].

In the polyurethane synthesis, an extensive variety of diisocyanates might be employed, including toluene diisocyanate (TDI), 4,4′-methylene diphenyl diisocyanate (MDI), 1,6-hexamethylene diisocyanate (HMDI) and isophorone diisocyanate (IPDI), among others [19]. Moreover, although traditional polyols consist of polyether or polyester, some approaches have been made to replace this source of hydroxyl groups by eco-friendly materials, for instance natural oils [2,24], or biopolymers [25,26]. In particular, castor oil [20,27] and cellulose derivatives [28,29] have been proposed as eco-friendly polyols. Vegetable oils are considered one of the most important types of renewable feed stock for the petroleum-based industrial products due to their noteworthy and favourable properties, since they are a copious resource, biodegradable, inexpensive, sustainable, non-toxic, easy to handle, structurally versatile, highly pure, flexible for chemical transformations, high physically and chemically stable, etc. [30,31]. Polyurethane production from vegetable oils is not a new approach. In fact, a wide range of natural oils have been considered as feasible choices to synthesized segmented polyurethanes [32,33], such as linseed or rapeseed [34], sunflower [35], canola [36], but principally soybean [29] and castor oil [14,15]. Castor oil belongs to the vegetable oil minority that exhibits bearing hydroxyl group [15], which, along with double bond presence [37], makes it an appealing resource for producing polyurethanes. In particular, some castor oil-based polyurethane formulations have been previously proposed as eco-friendly solventless adhesives [20,38]. On the other hand, due to the vast abundance of cellulose in nature, this carbohydrate and its derivatives have also drawn great interest in industrial production. For instance, different cellulose derivatives, i.e., methylcellulose, α-cellulose, 2-hydroxyethylcellulose, methyl 2-hydroxyethylcellulose and cellulose acetate propionate have been successfully employed in previous investigations [28], promoting the reaction between the hydroxyl groups located in the cellulose backbone and active diisocyanate crosslinkers to synthesize functionalized biopolymers which were further dispersed in vegetable oils. This study demonstrated that the rheological response, and therefore the application field, of the different oleogels achieved basically depends on the balance between the polarity and the size of cellulose substituents.

In the present work, novel bio-based polyurethane adhesive formulations have been prepared in two steps by combining a carbohydrate polymer like cellulose acetate and castor oil. Cellulose acetate was first functionalized by inducing the reaction with 1,6-hexamethylene diisocyanate. Then, the resulting product was blended with different proportions of castor oil producing bio-based polyurethanes with adhesion properties. The main objective of this work was to evaluate the influence of the castor oil/functionalized biopolymer ratio on the rheological, thermal and adhesion performance properties of these eco-friendly formulations.

## 2. Materials and Methods

Materials. The raw material used to synthesize the biopolymers was cellulose acetate (CA, 40% acetyl groups, Mr, ~29,000), which was modified by inducing the reaction with 1,6-hexamethylene diisocyanate (HMDI, purity ≥98.0%) using toluene (purum grade ≥99.7%) as solvent, and triethylamine (TEA, purum grade of 99.5%) as a catalyst. All these compounds were supplied by Sigma-Aldrich (St. Louis, MO, USA). Moreover, castor oil purchased from Guinama (Valencia, Spain) was used to prepare the bio-based polyurethane together with the modified cellulose acetate. Poplar wood, polyester fabric and sycamore wooden sheets were employed as substrates and supplied by a local store. U-Bond^®^ 309-TFC was selected as a commercial polyurethane-based adhesive and used as benchmark in the mechanical tests.

Synthesis of the bio-based polyurethanes. A two-step synthesis protocol was followed. Cellulose acetate functionalization was first performed following the protocol detailed in previous studies [39]. In the first step, the solvent was introduced into the flask and bubbled for half an hour with Argon, and then 15 g of CA, along with TEA and HMDI, were introduced into the flask in a molar proportion of 4.53:1:1 NCO:OH:TEA respectively. These reagents were vigorously mixed for 24 h at room temperature and under inert atmosphere. Finally, the solvent was removed from the mixture by means of a vacuum evaporation process, obtaining the functionalized biopolymer. Such a high NCO:OH molar ratio, i.e., excess of HMDI, and reaction time ensure the complete functionalization of available hydroxyl groups in cellulose acetate, as will be discussed below in Section 3.1.

Afterwards, the functionalized biopolymer and castor oil were placed together in an open vessel and blended using a controlled-rotational speed mixing device (RW 20, Ika, Staufen, Germany) provided with an anchor impeller. Castor oil/biopolymer ratio was varied, producing formulations with 20:80, 50:50 and 70:30 weight ratios. The excess of HMDI added in the CA functionalization step was estimated to theoretically counterbalance the amount of hydroxyl groups available for the highest castor oil/biopolymer weight ratio (70:30). These mixtures were agitated during 24 h, at room temperature. Afterwards, the resulting products were homogenized by using a rotor-stator turbine Ultra-Turrax T50 (Ika, Staufen, Germany), at 10,000 rpm, for 60 s.

Experimental Techniques. Bio-based polyurethanes studied were chemically, thermal and mechanically characterized by means of Fourier transform infrared (FTIR) spectroscopy, thermogravimetric analysis (TGA), differential scanning calorimetry (DSC) and rheological techniques as well as standardized mechanical testing. Characterization was carried out on cured samples one week after their preparation. After one week, properties remain unaltered.

The chemical structure of the synthesized polyurethanes was analyzed by FTIR spectroscopy in a FT/IR-4200 Spectrometer apparatus (JASCO Inc., Tokyo, Japan), equipped with an attenuated total reflectance (ATR) accessory provided with monolithic diamond crystal. Spectra were the result of an average of 73 scans with a resolution of 4 cm^−1^ and a 45° angle of incidence, in the spectral range 7800–350 cm^−1^.

TGA measurements were also carried out by using a Q50 Thermogravimetric Analyzer (TA Instruments, New Castle, DE, USA). Ten to twenty milligrams of each sample were heated up from 30 up to 600 °C at a heating rate of 10 °C/min, under nitrogen environment (flow rate, 60 mL/min).

DSC analysis over the cured polyurethanes was performed by using a Q100 Calorimeter (TA Instruments, New Castle, DE, USA). Five to ten milligrams of each sample were placed on aluminum pans and submitted to a heating cycle from −85 up to 250 °C at a heating rate of 10 °C/min, under inert atmosphere of nitrogen (flow rate, 50 mL/min).

The rheological characterization of bio-based polyurethanes was carried out using a controlled-stress rheometer Physica MCR301 (Anton Paar Germany GmbH, Ostfildern, Germany). Small-amplitude oscillatory torsion tests were performed within the linear viscoelastic region, in a frequency range 0.01–100 rad/s at different constant temperatures ranging from 25 up to 200 °C. The linear viscoelastic range was previously determined by performing stress sweep tests at 1 Hz. Furthermore, upward temperature ramps of 2 °C/min were applied from 25–200 °C at 1 Hz.

Mechanical properties for bonding wood were studied by means of Standard Test Methods ASTM D903, D906 and D1184, determining the stripping, shear and flexural strengths of the synthesized eco-friendly polyurethanes, using a Universal Testing Machine (Shimadzu, Kyoto, Japan), model AG-IS. Peeling strengths in poplar wood–polyester fabric joints (105 × 18 × 1 mm^3^ bonding line) were evaluated by applying a constant separation rate of 152.4 mm/min at room conditions, thus obtaining the stripping strength as the arithmetic average load per millimeter of width. On the other hand, poplar wood-to-poplar wood lap-shear strengths (18 × 18 × 0.5 mm^3^ adhesive volume) were obtained as the average maximum load per square centimeter of shear area when applying a force ramp of 3.56 kN/min. Finally, sycamore-adhesive bonded laminated assemblies, comprised of eight 38 × 19 × 0.25 mm^3^ plies, underwent a 3-point flexural test, with a crosshead speed of 0.74 mm/min, and associated flexural strengths were calculated according to ASTM D1184 (ASTM International, West Conshohocken, PA, USA). All substrates were cut according to the required dimensions, joined without applying a permanent pressure or any other pretreatment, and were kept at room temperature and 50% relative humidity for 7 days, according to these test methods. All data were analyzed by means of statistical tests to identify and remove possible outliers (Grubbs and Generalized Extreme Studentized Deviate (ESD) tests).

## 3. Results and Discussion

### 3.1. Chemical and Thermal Characterization

**Figure 1:**
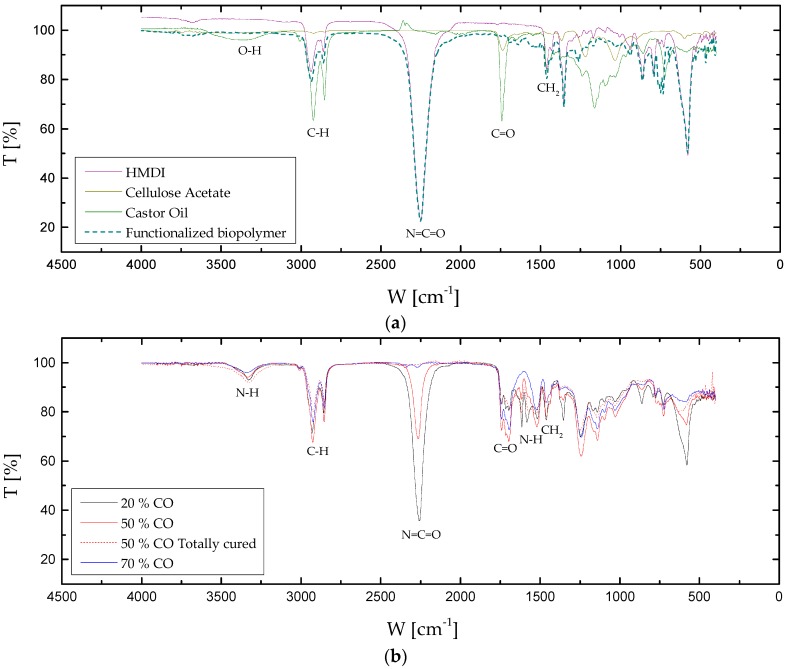
Fourier transform infrared spectroscopic attenuated total reflectance (FTIR-ATR) spectra for: (**a**) 1,6-hexamethylene diisocyanate, cellulose acetate, castor oil and functionalized biopolymer; (**b**) formulation with 20% CO, 50% CO, 70% CO and 50% CO totally cured.

The Fourier transform infrared spectroscopic attenuated total reflectance (FTIR-ATR) technique was used to evaluate the preparation of polyurethanes with different castor oil/biopolymer weight ratio. Figure 1 shows the infrared spectra for castor oil/cellulose acetate-based polyurethanes after seven days of curing (Figure 1b), in comparison to those obtained with the raw materials, i.e., 1,6-hexamethylene diisocyanate, cellulose acetate and castor oil (Figure 1a). Figure 1a also includes the FTIR spectrum of the functionalized biopolymer which was further blended with castor oil to get the final bio-based polyurethanes. As can be seen, the characteristic peak attributed to the O–H stretching vibration has almost disappeared by around 3330 cm^−1^, thus confirming the total functionalization of cellulose acetate as intended. On the other hand, bio-based polyurethanes (Figure 1b) also exhibit the absorption band centered at 3330 cm^−1^ due, in this case, to both the O–H and N–H stretching vibrations [23,40,41,42,43] as well as the band at 1741 cm^−1^ corresponding to the aliphatic carbonyl group (C=O) [40], which are in concordance with those found in the castor oil spectrum (Figure 1a). Besides, the absorption bands at 2919–2931 and 2851–2860 cm^−1^ correspond to the C–H asymmetric and symmetric stretching vibration, respectively, due to existing methylene groups in the polyurethane backbones, which can be observed in all IR spectra. Those peaks are accompanied by the bands at 1462 cm^−1^, corresponding to CH_2_ bending vibration [40].

Furthermore, the almost disappearance in the peak intensity at approximately 2260 cm^−1^, attributable to free N=C=O groups (Figure 1b) [23,39,40], confirms the complete reaction of N=C=O groups for the sample with the lower biopolymer content (Figure 1b), due to the excess of hydroxyl groups. This fact is corroborated by means of the reduction in the intensity of peak located at 1354 cm^−1^, attributable to C–N stretching vibration in free isocyanate groups. However, the intensity of the peak associated to the stretching vibration of the N=C=O group clearly increases as biopolymer content does. Moreover, the extensive formation of urethane linkages is confirmed by their characteristic bands apparent at 3330 cm^−1^, which appears to overlap the O–H stretching vibration but as a sharper peak, 1715, 1697 and 1580 cm^−1^ attributed to N–H stretching, amorphous and crystalline hydrogen bonded C=O stretching, and N–H bending vibrations, respectively [31,40,41]. Finally, no evidence of free isocyanate groups was found after one month of curing, as illustrated in Figure 1b for the 50:50 CO/biopolymer weight ratio. However, despite the fact that total curing process requires around one month, the bio-polyurethane mechanical properties remain almost unchanged after one week.

**Figure 2:**
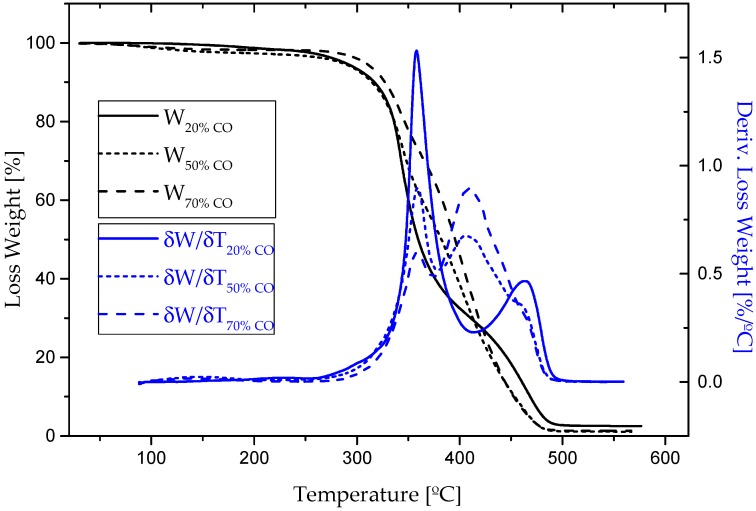
Thermogravimetric analysis (TGA) analysis for all bio-based adhesives.

**Figure 3:**
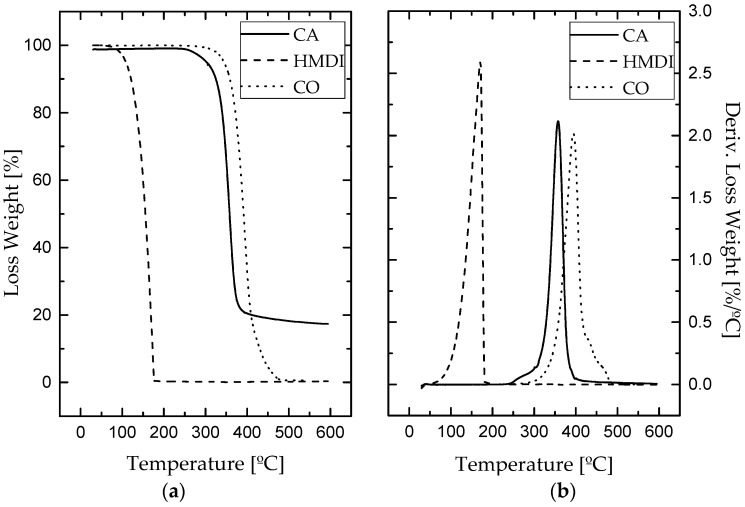
(**a**) Loss weight and (**b**) derivative loss weight curves for cellulose acetate, 1,6-hexamethylene diisocyanate (HMDI) and CO raw materials.

The thermal stability and decomposition behavior of bio-based adhesives were studied with the aid of thermogravimetric analysis after one week of curing. Figure 2 displays TGA curves for the different formulations studied, showing the weight loss percentage and the derivative curve versus temperature. Those results were also compared to those obtained with the corresponding reactants, i.e., cellulose acetate, 1,6-hexamethylene diisocyanate and castor oil (Figure 3). Table 1 collects the characteristic thermal parameters determined from the thermograms, such as, the onset temperature (*T*_onset_), the temperature for the maximum decomposition rate (*T*_max_) and the final temperature in each decomposition step (*T*_final_), along with the weight-loss percentage corresponding to each step and the percentage of non-degraded residue (R) at the end of the process.

For cellulose acetate, thermal decomposition under nitrogen atmosphere took place in one single step, as can be deduced from Figure 3, ranging from 335 to 373 °C, yielding an almost complete weight loss (≈82%) after this temperature. In comparison to pure cellulose acetate, bio-based polyurethanes prepared from this polymer experienced a slight decrease in thermal stability as a consequence of the polymerization process. As can be seen in Table 1, all synthesized bio-based polyurethanes generally start to decompose at lower temperatures, experiencing a not very significant weight loss (≈1–2%) within the range of 60–204 °C, which can be attributed to the loss of some free remaining NCO segments [44], but also residual solvent and moisture. Moreover, as a result of the urethane linkages, the biopolymer degradation started at lower temperatures. As previously reported, the isocyanate functionalization of cellulose derivatives generally expands the degradation temperature range [28,44].

Besides this, for castor oil, a single step thermal decomposition took place between 367 and 416 °C (see Figure 3), which is clearly noticeable in the thermograms of bio-polyurethane samples containing 50% and 70% CO (Figure 2). Interestingly, the formulation with 20% CO did not show this degradation peak due to the low oil content in its structure. As a consequence, the lower castor oil content, the less intense peak appeared at around 395 °C, and also the higher intensity in the event located at 343 °C, corresponding to cellulose acetate, was observed.

Finally, the bio-adhesive with the highest biopolymer content exhibited a clear peak centered at 465 °C, which does not correspond to the degradation of urethane groups. According to Gurunathan et al. [31,45] and Corcuera et al. [41], this event takes place at around 320–370 °C, overlapped in this case with the degradation of cellulose acetate backbone. At around that temperature (450–470 °C), a shoulder in the last decomposition peak was also detected in samples containing 50% and 70% CO as well (Figure 2). As a consequence, the degradation peaks found at these temperatures in the bio-based polyurethanes studied must be assigned to excessively crosslinked polyurethanes networks. These more rigid domains, logically favoured by the higher biopolymer content, i.e., higher density of urethane linkages, are more thermally stable [25].

**Figure 4:**
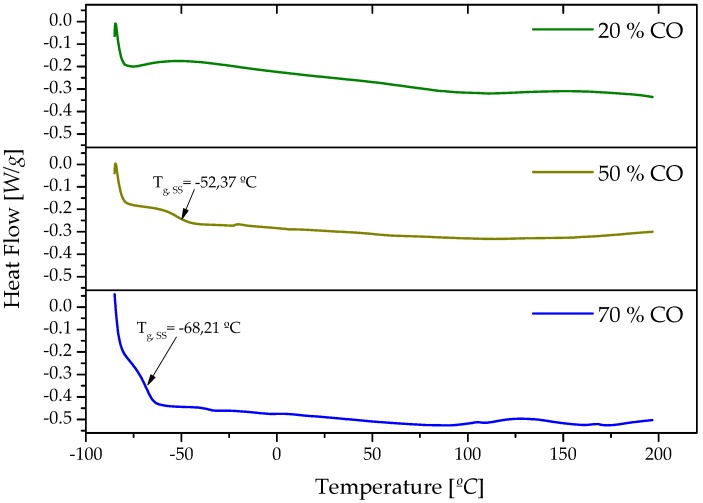
Differential scanning calorimetry (DSC) thermograms for bio-based polyurethane samples studied.

The different structural thermal events taking place in the synthesized cellulose-based polyurethane were evaluated by means of differential scanning calorimetry (DSC). Generally, only one thermal event, corresponding to the glass transition of soft segments domains (*T*_g,SS_), was detected in the temperature range applied (Figure 4), whereas no glass transition attributable to hard segments was observed [46]. Moreover, melting transitions of the crystalline domains were also not observed below 200 °C. The temperature of the glass transition values (*T*_g,SS_) were taken at the midpoint of the transition, and inserted in Figure 4. According to these results, *T*_g,SS_ increases with the biopolymer content in the formulation. Nevertheless, because of the low vegetable oil content, *T*_g,SS_ became unnoticeable in the formulation with the 20% CO content.

### 3.2. Rheological Properties

**Figure 5:**
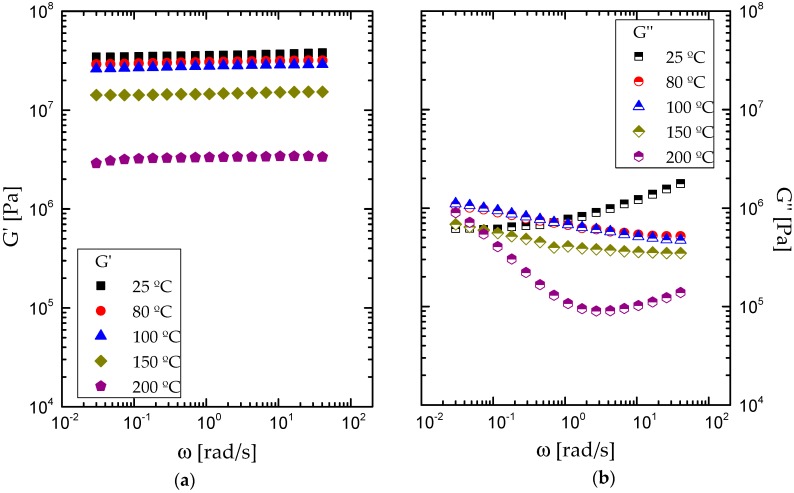
Evolution of (**a**) elastic, *G*′, and (**b**) viscous moduli, *G*″, with frequency, within the linear viscoelastic range, for formulations containing 50% CO castor oil at different temperatures.

Figure 5 shows the evolution of viscoelastic moduli (*G*′ and *G*″) with frequency for the sample containing 50:50 CO/biopolymer weight ratio, at different selected temperatures, measured under oscillatory torsional deformations. As can be noticed, a well-developed plateau region of the mechanical spectrum was always obtained, independently of the temperature, characterized by a predominant elastic behavior throughout the whole frequency range studied. Moreover, *G*′ decreases with temperature, slightly up to 80–100 °C and then more dramatically. The same influence can be observed in *G*″ at medium and high frequencies. However, the most remarkable effect of temperature on the viscous modulus is the change in the frequency dependence.

**Figure 6:**
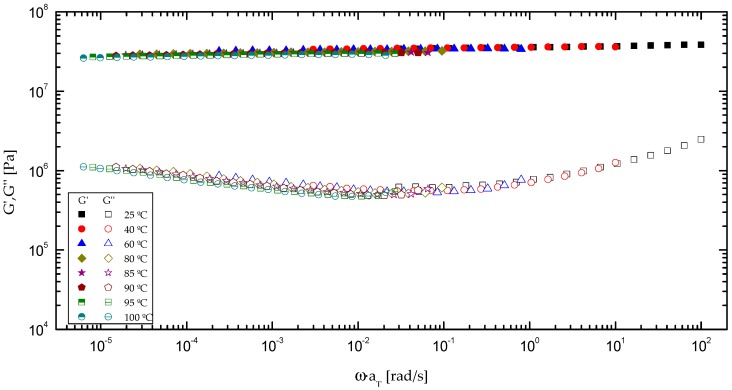
*t*–*T* superposition for formulation with 50% CO from 25 up to 100 °C temperature.

**Figure 7:**
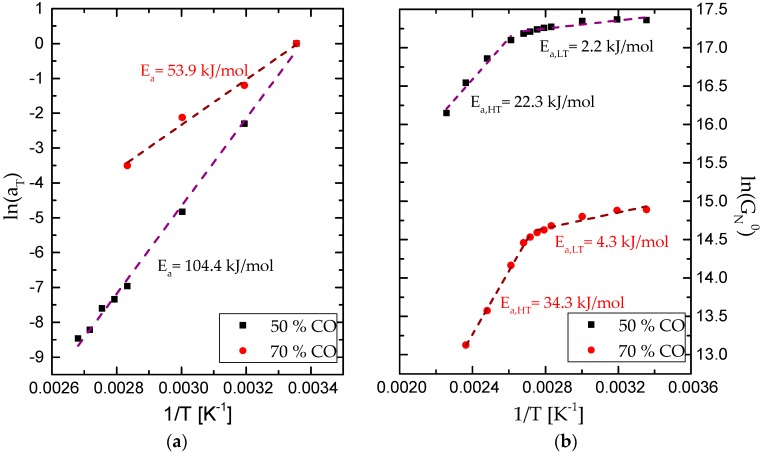
(**a**) Shift factors (*a*_T_) and (**b**) plateau moduli (*G*_N_^0^) for formulations with 50:50 and 70:30 castor oil/biopolymer weight ratios.

With the aim to extend the mechanical spectrum of the synthesized formulations, the time–temperature principle was applied (Figure 6), with the aid of suitable shift factors (*a*_T_). This principle can be properly applied to bioadhesives with 50:50 and 70:30 castor oil/biopolymer weight ratio, until approximately 100 and 80 °C, respectively, but no longer. Nonetheless, bio-based polyurethane with the lowest oil content (20%) showed an extreme brittleness, which hindered the torsional frequency response. The values of the empirical shift factors applied in the time–temperature principle are included in Figure 7a as a function of the reciprocal temperature, taking 25 °C as the reference temperature. The evolution of the shift factor with temperature can be described by means of the Arrhenius model as follow:

(1) $$a_{T}=A\cdot e^{\frac{E_{a}}{R}\cdot (\frac{1}{T}\;-\;\frac{1}{T_{0}})},$$

where *R* is the ideal gas constant (8.31434 J·mol^−1^·K^−1^), *T* the absolute temperature (K), *T*_0_ is the reference temperature (K), *A* is the pre-exponential factor and *E*_a_ is the activation energy (J·mol^−1^). Fitting values for the activation energy are shown in Figure 7a for each sample. As can be noticed, an increase in activation energy was found when CO content is reduced, meaning a bio-based adhesive characterized by a higher thermal susceptibility. The values of the plateau modulus, *G*_N_^0^, the characteristic parameter of the plateau region, estimated as the value of *G*′ at the frequency for which the loss tangent (tanδ = *G*″/*G*′) is minimum, were also plotted versus the reciprocal temperature (Figure 7b). As expected, *G*_N_^0^ values do not significantly change in the temperature range where the *t*–*T* superposition principle was applied, but dramatically decrease afterwards. The Arrhenius equation can be also applied to evaluate the temperature dependence on *G*_N_^0^ in the two above referred regions, below and above the critical temperature for a significant softening, as shown elsewhere [26]. Fitting values for the activation energy are included in Figure 7b. This behaviour reflects the thermo-rheological simplicity of those bio-based polyurethanes, from room temperature up to the mentioned critical temperatures, above which a different evolution of the rheological functions with both temperature and frequency was observed. This change in thermo-rheological response might be associated to the initial weight loss found in TGA tests, attributable to the loss of free isocyanate content.

**Figure 8:**
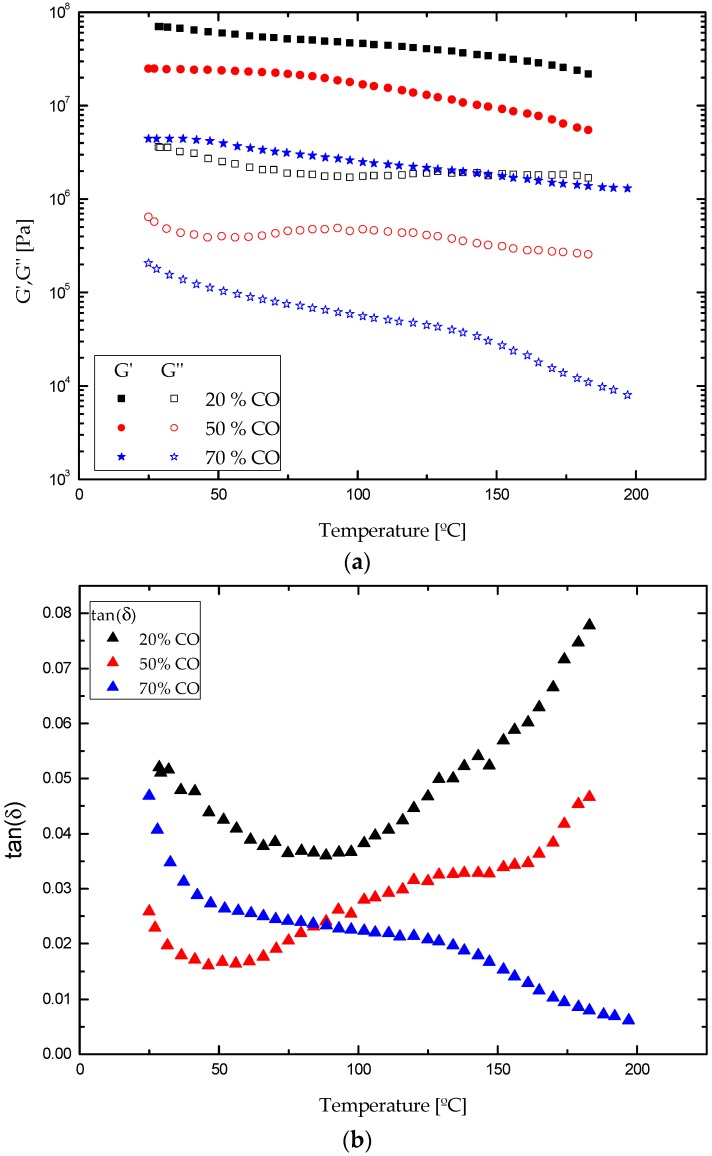
Temperature ramps for all ecofriendly bioadhesives at 2 °C/min of heating rate within the linear viscoelastic region: (**a**) elastic (*G*′) and viscous (*G*″) moduli; (**b**) loss tangent (tanδ).

In addition, Figure 8 depicts the evolution of viscoelastic functions when applying continuous temperature ramps for all formulations studied. Generally, in bio-based polyurethane systems with 20:80, 50:50 and 70:30 in castor oil/biopolymer weight ratios a slight temperature influence over the storage moduli (*G*′) was found up to a critical temperature of around 120, 80 and 40 °C, respectively. Above these temperatures, a more important softening was noticed, similarly to the evolution found in frequency sweep tests. The evolution of *G*″ is more complex since the frequency dependence is also affected by temperature as previously discussed. Nevertheless, the sample with the highest castor oil content (or lower biopolymer concentration) undergoes a continuous and much more important decrease in both viscoelastic functions within all temperature ranges evaluated. Moreover, attending to the evolution of the loss tangent, bio-polyurethanes with 20% and 50% CO exhibit a well extended plateau region in the range from 25 to 180 °C, with increasing values related to the melting transition of the crystalline microdomains, which is expected to be found at higher temperatures. Overall, the higher biopolymer content in the formulation, the higher values of the viscoelastic moduli and critical temperature for the softening were found.

### 3.3. Adhesion Performance on Wood Substrates

Adhesion performance of the synthesized bio-based formulations was studied by applying standardized mechanical tests. Table 2 shows the different mechanical parameters obtained from these tests. According to these, polyurethane with a medium CO content exhibits more important peeling strength in comparison to the other samples, although still slightly lower than the commercial polyurethane used as benchmark. Moreover, concerning shear tests results, the adhesive with 20:80 and 50:50 castor oil/biopolymer weight ratio present comparable shear strengths, almost three times higher than the sample containing 70:30 castor oil/biopolymer weight ratio (Table 2). These samples also undergo a substrate failure, which seems to be a more appropriate and desirable sort of failure. The values of the shear strength provided by the ASTM D906 test are almost twice as those reported by Somani et al. [38] for castor oil-based adhesives prepared using aliphatic diisocyanates, and slightly higher than those reported by da Silva et al. [20] for castor oil-based adhesives including TDI as crosslinking agent. Moreover, the lap shear strength values for adhesives containing 20:80 and 50:50 castor oil/biopolymer weight ratios are very similar to that obtained with the commercial sample. Finally, as judged by flexural results, all formulations showed similar failing loads, even though, once again, the sample with the highest castor oil/biopolymer weight ratio exhibited a stress value which is significantly lower than those obtained with the rest of samples, while the commercial adhesive provided, in this case, a significantly lower flexural strength.

## 4. Conclusions

In this research, a cellulose derivative was first functionalized by inducing the reaction with 1,6-hexamethylene diisocyanate, and then the biopolymer was blended with castor oil to obtain bio-based polyurethane adhesives. The influence of the castor oil/biopolymer weight ratio in bio-based adhesive was evaluated with the aid of rheological, standardized mechanical, thermal and spectroscopic analysis. The results demonstrated that the formulation with medium castor oil/biopolymer weight ratio (50:50 % wt) showed more suitable mechanical properties with more desired type of failure in wood joints under the mechanical conditions studied, comparable to those exhibited by a well-known commercial polyurethane-based adhesive. The rheological response of all synthesized polyurethanes corresponds to strong crosslinked gels characterized by the plateau region of the mechanical spectrum. Moreover, the storage and loss moduli increased with biopolymer content. A slight decrease in viscoelastic moduli was detected with the increase in temperature, becoming more important above a critical temperature, which increases with the biopolymer/castor oil weight ratio. Below this critical temperature, a simple thermo-rheological response was found, being able to apply the *t*–*T* superposition principle. Finally, thermal analysis, supported by rheological and spectroscopic results, suggests a chemical structure based on soft and crystalline hard segments, characterized by a several-stage thermal decomposition pattern.

## Figures and Tables

**Table 1:** Thermal parameters obtained from thermogravimetric analysis curves.

Sample	*T*_onset_ [°C]	*T*_max_ [°C]	*T*_final_ [°C]	Weight loss [%]	Residue [%]
20% CO	130/326/426	193/343/465	204/372/493	1.6/67.5/28.5	2.6
50% CO	63/314/374	98/344/397	158/358/442	2.3/39.2/47.2	1.2
70% CO	60/307/370	93/343/402	155/357/453	1.6/26.9/63.1	1.4
HMDI	138	171	176	99.7	0.3
Cellulose Acetate	335	358	373	82	18
Castor Oil	367	395	416	99.4	0.6

**Table 2:** Peeling, shear and flexural strengths on bonding wood substrate.

Sample	Peeling strength [g–f/mm]	Shear strength [MPa]	Flexural strength [MPa]
20% CO	59.8 ± 10.9 ^a^	2.37 ± 0.03 ^b,c^	21.9 ± 6.0 ^d,e^
50% CO	169.4 ± 19.6 ^b^	2.84 ± 0.36 ^b,c^	21.6 ± 4.9 ^d,e^
70% CO	76.9 ± 4.9 ^b^	0.94 ± 0.24 ^b^	14.5 ± 0.9 ^d^
Commercial polyurethane adhesive	228.6 ± 28.3 ^b^	2.50 ± 0.40 ^b^	11.0 ± 3.0 ^d,e^

Failure classification: ^a^ Adhesion; ^b^ Cohesion; ^c^ Substrate; ^d^ Breaking; ^e^ Buckling.
